# Hysteria and Organic Disease

**Published:** 1890-04-05

**Authors:** 


### The Practitioner's Mirror and Retrospect.

[*The Editor will be glad to receive offers of co-operation and contributions from members of the profession. There is no doubt that much valuable material full of interest and instruction is at present lost to science. This is largely so in the case of* (1) *the younger and less-known members of the hospital staff* (2) *those connected with cottage hospitals, and* (3) *the great body of medical practitioners not attached to any institution. The co-operation of all is cordially invited to enable the Editor to make* The Hospital *of the utmost possible use and value to the profession. Specialists willing to review books on their special subjects are invited to communicate this fact at once. All letters should be addressed to* The Editor, The Lodge, Porchester Square, London, W.]

### MEDICINE.

### HYSTERIA AND ORGANIC DISEASE.

We have been accustomed to accord to hysteria the power of simulating all kinds of organic disease, but now Dr. Buzzard has given an address at the annual meeting of the Neurological Society, "On the simulation of hysteria by organic disease of the nervous system." Dr. Buzzard says there are few cases of disseminated sclerosis in females in which marked hysterical symptoms are not mixed up with those belonging essentially to the disease. This combination causes a liability to mistakes of diagnosis. A sudden or gradual loss of power of a limb in an apparently healthy young female; a localised numbness, or "pins-and-needles" sensation are symptoms, familiar enough, of functional trouble. But they represent modes also in which organic disease may make its appearance. But hysterical paralysis is most often complete, while the loss of power in disseminated sclerosis is very rarely more than moderate, except in advanced cases. The hysterical woman who has lost all power in her legs will very often, later on, lose the power of one arm; but Dr. Buzzard has not found that she is prone to lose the power in a limb, then recover it, and then lose it in another. It seems to Dr. Buzzard that the idea of this shifting powerlessness being suggestive of hysteria, has arisen from mistakes in diagnosing, as hysteria, cases of disseminated sclerosis. The shifting about of a state of more or less powerlessness appears to be *sui generis*, and should save us from error. Similarly, with regard to shifting impairment of vision. When the ophthalmoscope shows atrophy of disc, experience teaches that a diagnosis of functional disorder must be discarded. Many symptoms which have come to be considered characteristic of hysteria, will, if examined by the light of improved knowledge and experience be relegated to disseminated sclerosis. It appears to Dr. Buzzard that there is a connection of some kind between the two affections, but what that connection is, he is at present unable to state.

